# Selections

**Published:** 1816-08

**Authors:** 


### PART III.

### SELECTIONS.

### FOREIGN WORKS ON MEDICAL JURISPRUDENCE.

( Concluded from p. 70. )

#### *Medico-legal Examination of Bodies.*

3. *Dissection.*—It is, perhaps, of little consequence as to the part or cavity at which the examination is commenced. If any external wound or other injury exist, it is customary to begin there; and care should be taken so to direct the necessary incisions that they may not implicate the lesion. In other cases, the priority of inspection may be decided by the evident or suspected nature of the accident, or other existing circumstances. Thus, in death from apoplexy, or any cerebral commotion, the head may be first examined; in asphyxia or infanticide, the chest; in death from poison, the abdomen. But we here repeat, and may every medical practitioner bear it constantly and steadily in remembrance, that, in no case, and under no circumstances, ought one of these cavities to remain unexplored. In our description, the regular order will be pursued.

*Head and neck. Cranium.*—After dividing the scalp, and detaching it in the usual manner, the cranial bones should be cautiously examined, so that no fracture, fissure, separation of the sutures, depression, or other injury, inflicted on them, may escape notice.* All due precautions should be taken to distinguish sutures from fracture; considerable deviations and varieties sometimes existing in the course or aspect of the former. If any lesion be discovered, we are next to determine, by investigating the state of the soft parts, whether it were inflicted before or after death. The calvaria is next to be removed, by sawing the bones in a circular direction. Great patience and delicacy are requisite in this part of the operation, to avoid injury of the internal parts. The circumstances particularly to be remembered here, are, the inequalities, in thickness, of the cranial bones; the existence of the internal frontal spine and internal occipital ridge, which will generally require the saw to penetrate deeper at the corresponding portions of the circle than at any other; and the firm adhesion commonly connecting the internal surface of the calvaria to the dura mater: it is particularly strong in young subjects; and, for the most part, indicative of no morbid state. To diminish the difficulties attendant on this separation, another mode of operating has recently been proposed. This consists in removing by the trephine four circles of bone from the cranium, —two anteriorly, right and left, from the parietal (coronal) border of the frontal bone; and two posteriorly from the mastoid (posterior inferior) angles of the parietal; detaching the dura mater by the introduction of a flexible spatula through the holes thus formed; and afterwards sawing the cranium in a circular direction from hole to hole. The calvaria removed, we are cautiously to examine whether there be any exostosis or excrescence on its internal surface;—next, the state of the membranes,—of the vessels, whether turgid, empty, or ossified; whether the membranes display traces of inflammation, morbid adhesion, ulceration, fungus, collections of blood, lymph, or pus; whether ruptured, detached, pierced by osseous splints, or otherwise wounded. In inspecting the brain itself, it is highly important to employ a good scalpel, to remove its substance by horizontal sections in very thin slices, and deliberately examine each slice in succession; so that no tumor, no collection of blood or matter, seated in the internal structure, may, however small, escape detection. The general state and appearances of the cerebral mass noted, as to the existence of mechanical injury or mor-

---

* Absence of apparent injury of the cranial integuments, in cases of death from external violence, should never be admitted as proof that the skull and contained organs are not implicated in the mischief;—or set up as an apology for neglect or indolence in the examination In one of the most dreadful cases of cranial fracture, depression, and deep-seated cerebral injury which it has ever been our lot to witness, the scalp no where exhibited the slightest mark of violence.—Editors.

bid condition; the parts demanding particular notice, are, in the centre, the two lateral ventricles, their contained fluid, the plexus choroides, &c. their reflected and posterior horns should not be forgotten; the other three ventricles; the pineal gland and tubercula quadrigemina, and internal structure of the cerebellum;—in the base, the origin and condition of the cerebral nerves, medulla oblongata; pituitary gland, blood-vessels, as internal carotid and basilar arteries, jugular veins, and numerous sinuses. The whole of the brain should be removed, that the orbitar plates of the frontal bone, and whole basis cranii, may be minutely inspected, and no perforation, fracture, exostosis,* or other morbid phænomenon, pass unobserved.

*Cavity of the mouth, and organs of the cervical region.*—The head of the subject should be so placed, that the anterior part of the neck may be rendered perfectly tense. This may be conveniently done by placing a high block beneath the shoulders, and suffering the head to fall back unsupported. A longitudinal incision in the course of the median line is then to be made through the substance of the lower lip, and carried downward to the superior margin of the sternum; and another following the basis of the inferior jaw; the skin and platysma myoides reflected to the lateral parts of the neck; the jaw bone sawn asunder at the symphisis; and the parts adherent to its internal surface detached. Drawing down the tongue, we now come to the isthmus faucium; and, dividing the pillars of the fauces, expose the whole extent of the pharynx. The œsophagus and adjacent organs will be found by prolonging the incision downward and on each side. But to examine them properly, it is requisite that the sternum be previously removed; and, with a view of more fully exposing the bronchia and large blood-vessels at the root of the neck, part of the sternal extremities of the clavicle and first rib should be taken away with the sternum, on each side. In inspecting the air-passages, we first detach with caution the thyroid gland, and lay open the trachea and larynx by a longitudinal incision from the bronchia to the hyoid bone. The points here requiring particular attention are, with respect to the mouth, whether it exhibit any marks of violence, or contain extraneous substances; the general state of the constituent bones and investing membrane; the tongue† and more important glands, as the sublingual and tonsillar; the various orifices communicating with this cavity, as the posterior nares, Eustachian tubes, rima glottidis, œsophagus;—and the epiglottis:—

---

* We have known fatal epilepsy determined by an osseous tumor seated on the cerebral surface of the orbitar plate of the frontal bone. The anatomical collection of the late Mr. Allan Burns, of Glasgow, contains a valuable specimen of this kind.—Editors.

† Children, in whom the frenum of the tongue has been improperly divided, have died of suffocation, consequent on introversion of that organ, and obstruction of the glottis by its apex.—Editors.

with respect to the neck, the various glands, the parotid, submaxillary, thyroid; the alimentary and aërial tubes, their parietes, and canal, whether pervious or obstructed by extraneous matter, or the products of disease, and particularly the state of their mucous membranes; the blood-vessels, common carotid arteries, their internal and external divisions, the primary branches of the latter, the subclavians at their origin, and branches to the thyroid gland and other parts of the cervical region; the external, internal jugular and subclavian veins; the termination of the thoracic duct at the angle formed by the two latter, on the left side; the more important nerves, as the par vagum, and its superior laryngeal and recurrent branches, especially the trunk itself, situated between the carotid artery and internal jugular vein, posteriorly; the great sympathetic, more closely connected with the spine, and distinguishable by its ganglions, the phrenic, lying on the anterior scalenus muscle; and the commencement of the great brachial plexus, passing behind that muscle to the arm. This too will be the proper time to examine the cervical portion of the vertebral column. The bodies of the vertebræ will be most advantageously inspected in that situation of the subject which we have before described; but, in exploring the state of the processes, posterior ligaments and spinal marrow, we must change the position of the body, dissect back the integuments from the posterior cervical region, and detach the muscles from each side of the spinous processes. After determining the existence of any osseous lesion, morbid or mechanical, as caries, fracture, dislocation, or rupture of the ligaments, the spinous processes may be sawn off, whereby the spinal marrow will be exposed. The condition of it and the enveloping membranes is carefully to be noted, with respect to any mechanical injury, induration, inflammation, or effusion of blood, lymph, or purulent matter. The atlas, dentata, its tooth-like process and their ligaments, should be minutely inspected; the vertebral arteries, mounting up through the foramina of the transverse processes of the cervical vertebræ; and the cervical nerves, on their exit from the foramina between. At this period also, the remnant of the column may be conveniently explored.

*Thorax.*—A longitudinal incision should be made from the top of the sternum to the base of the ensiform cartilage; another transverse, along the clavicles, and terminating at their acromial extremities, and a third along the cartilaginous margins of the ribs to the projecting point of the fourth false one. The integuments and muscles are then to be reflected, and the sternum to be raised by cautiously dividing the cartilages of the ribs at their junction with the bone, by means of a saw or strong scalpel, so that the contained viscera may not be injured.* Ere putting aside the ster-

* We have known aukward or inexperienced dissectors, mistake for the consequences of previous violence or disease, incisions of the internal organs, made by their own scalpels. Of the proposal of Chaussier, to commence the examination of the chest by removing the dorsal portion of the spine, we are unable to decide the merits by experience.—Edit.

num, we examine it as to the existence of caries or fracture; and the anterior mediastinum, whether it contain any effused fluid, or display traces of inflammation, and what, in the child, is the state of the thymus gland, and also of the ribs. We now view the general condition of the thoracic cavities, whether there be wound, inflammation, or adhesion of the costal and pulmonary pleura, or effusion of any kind between them. If any wound, penetrating the substance of the lungs, be suspected to exist, its precise site may be determined by inflating the trachea. The practitioner should be reminded, not to mistake, for the effects of disease, that dark purple mottled appearance of the lungs, commonly observed in persons of advanced age, or their liver-like colour and consistence, in the more dependent situations, merely produced by the natural tendency of the blood to gravitate. After opening the primary ramifications of the bronchia, and ascertaining the state of the bronchial glands,* the right lung may be cautiously turned out, in order to get at the posterior mediastinum, and examine the important organs therein contained. These are the œsophagus, the par vagum, the left nerve of which lies on the fore-part of the œsophagus; the right, on its back part; the aorta, posteriorly, but somewhat to the right; the vena azygos, still farther to the right; the thoracic duct, between them; and the anterior intercostal nerves. The trunk of the intercostal will be seen running down on the heads of the ribs, and forming ganglions in the intercostal spaces. The condition of all these, as to any wound, rupture, inflammation, thickening, dilatation, obstruction, effusion of blood, serum, pus, or chyle, should be minutely noted. For more close and convenient inspection, the thoracic organs may now be removed, the quantity of any fluid, effused into their containing cavity, having been previously determined, and tight ligatures passed round the great cervical vessels above, and the inferior vena cava, aorta, and œsophagus, below. On opening the pericardium, we should remark whether it exhibit any wound, adhesion externally to the surrounding parts, or internally to the heart, the extent and nature of such adhesion; whether inflammation, thickening; the amount and qualities of any effused fluid which it may contain. The heart itself may now be inspected. After remarking any external peculiarities of position, size, colour, or appearance, we expose the cavity of the right auricle by an incision carried from the superior to the inferior vena cava; then that of the corresponding ventricle,

* The examiner will bear in mind, that these bodies, independently of any morbid action, commonly contain a black ink-like fluid. We once witnessed a ludicrous scene among some medical gentlemen ignorant of this fact: one of whom, more great and "experienced" than the rest, would, doubtless, have published an ingenious "Memoir on this wonderful phænomenon," had not we, with our wonted charity, interfered in a seasonable moment to rescue him from the ridicule and disgrace which he would, otherwise, have inconsciously drawn upon himself.—Edit.

by another incision through the auriculo-ventricular orifice to its apex: a third, directed obliquely to the right from the apex, will lead to the origin of the pulmonary artery. When we have noted the general condition of the parietes of this side of the heart, whether thickened, wasted, ruptured, displaying abscess or induration; of the internal membrane, whether inflamed, thickened, ulcerated, cartilaginous, ossified, forming fungus or other tumor; of the cavities, whether dilated, contracted, gorged with blood, or empty, the quantity and physical properties of such blood; whether containing pus, or any osseous or cartilaginous body or fibrinous concretion; the other parts, requiring minute attention, are the various orifices and their valves, as the foramen ovale, orifices of the coronary vein, of the ventricle, and pulmonary artery; whether these orifices are enlarged or contracted, whether their valves mal-formed, ruptured, inflamed, indurated, ossified, ulcerated, giving rise to fungous excrescences, or otherwise fit to perform their functions; the carneæ columnæ and their tendinous chords, whether ruptured or otherwise diseased. The left, or aortic side of the heart, should be explored with the same minuteness, beginning by incision of two of the pulmonary veins, and terminating at the aorta. The parietes of this portion, it should be remembered, are naturally thicker than those of the right. The aorta, its capacity, valves, coronary arteries, state of its internal membrane, and of the three great vessels arising from its arch, must also be inspected, and any malformation or morbid appearance rigorously described. In cases of supposed infanticide, it will also be necessary to observe whether or not the canalis arteriosus, connecting the aorta and pulmonary artery be pervious. The internal structure of the lungs next demands notice; whether universally crepitous, whether gorged with blood or empty, the colour and consistence of the blood; whether the bronchial tubes contain water, mucus, blood, pus, or other foreign liquid; whether the vascular structure displays inflammation, induration, tubercle, abscess, gangrene; and, for this purpose, the organ should be cut in very thin slices. Of the conduct to be pursued in determining the question of infanticide by the hydrostatic trial of the lungs, it is unnecessary to speak, after the copious manner, in which we have recently discussed that interesting subject.* Before quitting the chest, we should now examine the state of the ribs, costal pleura, bodies of the dorsal vertebræ, and superior surface of the diaphragm; whether displaying wound, rupture, inflammation, ulceration, abscess; the condition of its orifices, whether naturally formed, contracted, or obstructed by any tumor so as to impede mechanically the functions of the inferior vena cava, aorta, or œsophagus.† Replacing the viscera of the thorax, we next proceed to

* See our first volume, p. 264—Edit.

† During our researches in comparative anatomy, we have, more than once, observed abscess in the substance of this muscle, particularly in the sheep. It is frequently found ruptured in sudden death from external violence.—Edit.

*The abdomen.*—Every external appearance of mechanical injury or disease, as wound, contusion, tumefaction, hernia, &c. should be first noted. The cavity is then to be exposed by an incision from the sternum to the umbilicus, and from thence, on each side, to the spine of the ilium. In doing this, we should employ great precaution not to wound the contained viscera; as strong adhesion often exists between them and the abdominal parietes. The state of the cellular membrane, muscles, adherent peritonæum, and relic of the umbilical vein,* may now be viewed. After investigating the general state of the abdominal cavity, as to regular disposition, colour, magnitude, adhesion of its various organs, and the presence of any effused fluid, serum, blood, pus, alimentary or fæcal matter, we commence the particular inspection of each viscus and division. Ligatures should be cautiously passed round the biliary ducts and commencement of the jejunum, as it emerges from beneath the omentum: thus the stomach and duodenum may be detached without losing their contents. We view their external aspect; slit them up the whole length, ascertain the nature of the inclosed matters, (which may be preserved or otherwise, according to circumstances), and clear the internal surface with a soft sponge. Other ligatures applied to the termination of the ilium, at the caput coli, and again to the colon as it descends into the pelvis, and the intestine divided in the interspace, we gently separate from their mesenteric and other membranous attachments, the two remaining portions of the alimentary tube, and examine their parietes and contents with the same precautions as the first.† We then inspect, in succession, the liver, spleen, pancreas, kidnies, with their capsules and ureters, mesentery and other productions of peritonæum, thoracic duct, and blood-vessels, the abdominal aorta, inferior cava, and their primary branches; and, in the new-born child, the canalis venosus, and lastly the nerves. The unnatural or morbid appearances which may be common to all these various organs, are wound, rupture, or division; enlargement or dilatation, diminution or contraction, sanguineous congestion, inflammation, adhesion, induration, tumor, scirrhus, hydatid, suppuration, ulceration, sphacelus. The particular circumstances requiring attention are, in the *stomach and intestines*, their contents, as worms, gall-stones, indurated fæces, intestinal concretions, poisonous or other extraneous substances; the state of their parietes, as to hernia, strangulation, intus-susception, thickening, extenuation, corrosion, perforation, stricture; of their orifices, the cardiac, pyloric, cysto-hepatic and ilio-colic, as to dilatation, straitening or obstruction.‡ In the *liver*, the degree of congestion with blood or

---

* Particularly in cases of infanticide. We lately found it pervious in a female subject of sixty years.—Edit.

† The method to be pursued in cases of death from poison is described in our review of Orfila's *Toxicologie*, page 423 of our first volume.—Ed.

‡ We would warn the examiner not to mistake a diffused pink blush, bile, condition of the biliary receptacle, ducts, and secretion; whether gall-stones or other obstruction exist. In the *pancreas*, whether the duct be pervious or lodge any concretion. In the *kidnies*, whether the pelvis and ureter be dilated, contracted, or choked by any urinary calculus. In the *mesentery*, the condition of its absorbent glands. The *thoracic duct*,* whether completely pervious from its origin. The *aorta*, *vena cava*, and their system of *abdominal blood-vessels*, whether dilated, aneurismal, ossified, contracted, or obliterated. The *nervous plexuses*, formed by the eighth pair and intercostal, and the trunks sent off from the lumbar region and sacrum to the lower limbs, whether indurated, softened, discoloured, or compressed by any neighbouring tumor. Lastly, we view the inferior surface of the diaphragm, bodies of the lumbar vertebræ, and cellular membrane in the vicinity of the psoas muscle, sometimes the seat of chronic abscess.

*The pelvic organs* yet remain to be examined. After taking a survey of them, *in situ*, it will be best to remove them for more minute inspection. This may be conveniently accomplished by dividing the pubis at the symphysis, detaching the bladder, rectum, &c. from their connections to the pelvic bones, practising externally an oval incision which may include the genitals and anus, and drawing out the viscera of the pelvis from beneath the pubic arch. The bladder, rectum, and, in the female, the uterus and its appendages, are the organs requiring inspection: and, independently of the mechanical and morbid lesions common to every form and variety of animal structure, we should note particularly the following circumstances:—In the *bladder*, the existence of any urinary concretion, or other foreign body; state of its parietes, as to thickening, extenuation, rupture of its ureteric and urethral orifices, of the prostate, vesiculæ seminales, vasa deferentia, urethra, scrotum, testes and spermatic cord. In the *rectum*, presence of extraneous matter, or marks denoting the perpetration of the foulest of human crimes. In the *uterus*, *ovaria*, and *Fallopian tubes*, vestiges of conception, abortion, recent delivery, hæmorrhage or polypus, state of the vagina and external parts, as to the hymen and other appearances which may indicate the recent act of sexual intercourse, or attempts at violation. Finally, we survey the bones, ligaments, and muscles, which enter into the composition of the pelvis; and, in cases of pregnancy, determine

---

sometimes observed on the villous surface of the stomach, and of obscure origin, for the consequences of inflammation; or the yellow or green discolouration of the duodenum, colon, &c. caused by transudation of bile, for the sign of disease or impending disorganization.—EDIT.

* Inspection of this important little vessel is commonly slighted or forgotten; but the anatomical examiner should never neglect, in doubtful or obscure cases, to inform himself of its condition, by injecting it with air or other fluids.—EDIT.

correctly the various dimensions of this cavity; and thus settle the question whether or not delivery were practicable.

The observations of Bichat, on the colour of the mucous membranes, will form an appropriate close to this article. An accurate knowledge of their varieties in the natural state is essentially necessary to the pathologist. He "should never lose sight of the primitive tint of that portion of the mucous structure which he is examining; since each division of this system presents remarkable differences of shade. If the membrane of the bladder or rectum be as red as that of the stomach in its natural state, we may decide that there has been inflammation: if the redness of the sinuses equal that which is natural to the bladder and rectum, inflammation also exists there. For the mucous system, a scale of colouration is established; a precise knowledge of which should be acquired, as a standard to which we may refer the inflammatory state in dissections."*

We think it right to add, that, although the outline and materials of the preceding article have been principally drawn from the foreign works, specified in our last number, the results of our own practice and experience in anatomical research have been, sometimes, and the reader will perhaps think too largely, introduced.—EDITORS.

### FOREIGN JOURNALS.

TOXICOLOGY. *Case of Poisoning by Ammonia inspired during an attack of Epilepsy.*†

"A physician, aged 30, of strong constitution and sanguine temperament, had been, several years, subject to epileptic paroxysms, for which he had followed, during nine months, an empirical plan of treatment."

"One morning, after having breakfasted on chocolate, he had a paroxysm in the presence of his porter. This man, observing on the chimney-piece a small phial which contained ammonia, and presuming it to be the wonted remedy for the convulsions, repeatedly moistened with it the corner of a handkerchief which he applied to the nostrils of the patient, and introduced into his mouth. Two drachms of ammonia were thus expended. We may calculate, however, that one half only of this passed into the nostrils and mouth: and there is reason to suspect that the porter, from having frequently seen large doses of Hoffman's anodyne given to epileptics in the streets, might have attempted also to pour the alkali into the latter cavity."

* *Anatomie Generale,* tome iv. p. 464.

† *Observation rapportée par M. le Docteur Nysten. Gazette de Santé, May,* 1816. Instead of "*inspired,*" we should be disposed to say *introduced into the larynx and œsophagus.* The symptoms and dissection alike warrant this conclusion. EDITORS.

"However this be, the paroxysm was of long duration: and the patient, on recovering his consciousness, felt a burning pain from the mouth to the gastric region, and great tightness of respiration. He swallowed a grain of opium, and prescribed for himself a draught of kermes (sulphuret of antimony) of which, however, a small portion only was got down. Doctor Chrestien, who first visited the patient, found him in a state of irritation and extraordinary suffering; scarcely able to swallow, respiring with great difficulty; each act of inspiration attended by a kind of rattling. Leeches were applied to the throat without relief. An emulsion, directed for common drink, induced cough with copious expectoration of mucus. Dr. Nysten saw him at seven o'clock on the following morning. He had passed a sleepless night: the countenance was changed; respiration frequent, difficult, stertorous. A serous fluid oozed at times from the nasal cavities, which the air could no longer penetrate. The thirst was urgent; deglutition very difficult. The cough and expectoration of mucus were particularly excited by the passage of any liquid into the pharynx; and little of it penetrated the œsophagus. A pound at least of mucus, mixed with the emulsion, had been voided during the night. The voice was low, feeble; articulation fatiguing and interrupted, from the state of respiration. A small black slough was seen on the centre of the lower lip, and another on the summit of the tongue. The surface of this organ was white. The velum palati, faucial pillars, tonsils, and posterior wall of the pharynx, of a deep red. The uvula was retracted, and covered with a white mucous film: the tonsils little tumefied, The patient complained of burning heat in the throat, breast, and stomach. He had passed a little red urine. A chronic diarrhœa, which his empirical remedy had kept up, was now suppressed. The skin was warm and dry; pulse small, frequent, and feeble: intellectual faculties unimpaired. Dr. Nysten directed a large blister to be applied, as a revulsive, to the sternum, emollient injections, and continuance of the emulsion."

"Evening. Condition unaltered, except that the weakness was augmented. The patient, by means of a vessel furnished with a spout, swallowed some liquid, but little in comparison with his wants. His physicians then recommended perseverance in injections of veal-broth; but the fluid was repelled with force from the rectum at the moment of its introduction. The night was passed in undiminished suffering. The patient, perfectly conscious of his situation, abandoned himself to despair."

"Morning. Great weakness. The blister had detached the epidermis, but excited no serous secretion. Two others, applied in the vicinity of the first, produced no more effect. The extreme oppression, increase of the rattling, sense of suffocation, smallnes and depression of the pulse, now scarcely perceptible, — all announced the last impending struggle; yet the unfortunate man was in full possession of his senses: tormented with devouring thirst, he could get down but very little liquid. In the hope of at least affording him relief, Dr. Nysten introduced an elastic gum tube into the œsophagus, through the left nostril; and thus injected emulsion by a small syringe into the stomach. An unavailing attempt was also made to throw glysters up the rectum by means of a similar apparatus; the liquid was forcibly rejected, doubtless by a spasmodic contraction of the large intestines. At ten o'clock, the pulse was no longer perceptible: at eleven, the patient expired.

" *Dissection.* The membranes of the brain were sound, and merely exhibited some adhesions between the arachnoid, and the glands of Pacchioni, situated externally to the superior longitudinal sinus. The cerebral mass was injected, as in most sanguineous subjects. There were only some drops of serum in the lateral ventricles. The cornu ammonis, of the left side, was much firmer than that of the right, or the other parts bounding the ventricles. This firmness was particularly remarkable in the portion of the cornu ammonis which terminates at the digital cavity. The tuber annulare was also firmer than natural. The basis of the brain, and cerebellum, were perfectly sound. The mucous membrane of the nasal cavities was every where of an intensely red colour, and covered by an albuminous, membrane-like layer, which closed the nostrils anteriorily."

" The tongue presented no other alteration than the slough before mentioned. The mucous papillæ of its base were very large; the velum palati, its pillars, and whole membrane of the fauces, intensely red. The anterior surface of the epiglottis was sound, but the posterior, and the entrance of the glottis, very red, and covered by a false membrane. All the interior of the trachea and bronchia were of a bright red colour, and lined here and there with a membranous layer: portions of it were seen even in the bronchial tubes. The lungs were crepitous anteriorily: but posteriorly gorged with blood, probably gravitating there after death. The pericardium contained little serum. The heart, though somewhat voluminous, was healthy."

" The mucous membrane of the œsophagus presented some streaks of bright red; the same were seen in the stomach, following the course of the muscular fibres. The duodenum was sound. There existed a small intussusception about the middle of the jejunum. The mucous membrane of this intestine and the ileum presented several red patches: the large intestines were sound. The urinary bladder was much contracted, and displayed, towards its base,* vestiges of inflammation. All the remaining viscera were healthy."

" These facts, in the opinion of Dr. Nysten, prove that the pa-

* " Le trigone vésical" is the language of the original It means the triangular prominence, formed between the insertion of the ureters and prostate gland, by the ureteric muscles. See Mr. C. Bell's admirable description of these muscles, Medico-Chirurgical Transactions, Vol. III, page 171. Ed.

tient was destroyed by acute inflammation of the mucous membrane of the larynx and bronchia, induced by the ammonia, and analogous to acute croup. It was from the violence of the inflammatory action, and not from suffocation or asphyxia, that the patient perished. Vauquelin suggested that the prompt inspiration of an acid vapour, as that of the muriatic or acetic acid, might, in such a case, preserve the patient; but Dr. Nysten justly observes, that if the first moment (of the accident) be passed, and inflammation already developed, acid vapours can serve only to aggravate the symptoms. Under these circumstances, we must confine ourselves to the use of antiphologistic and evacuating remedies."

"A fact, recorded by Baron Percy, proves the destructive properties of the vapours of ammonia. A child, having climbed upon a foot-stool to reach a phial of this substance, fell, while holding it. The bottle brake; and the child, whose head was immersed in the ammoniacal vapour, died instantaneously from suffocation."

### *Case of poisoning by a mixture of sulphuric Acid and Indigo (bleu en liqueur.)**

"Mademoiselle A. D. aged seventeen, took, on the 26th of January last, two ounces of this compound, with a view of destroying herself. As soon as the first vomiting was excited, her sister sent for a physician. In the absence of my father, I visited the patient, whom I found with a red face and eyes somewhat haggard: she had also nervous attacks, and felt very severe pains in the stomach, I immediately prescribed a grain of emetic tartar in a glass of milk: some moments afterwards, she discharged a considerable quantity of the poison. A second grain of the emetic tartar, administered in a glass of water, induced repeated vomiting; but the ejected liquid, of which previously the colour had been blue, now assumed, from the admixture of bile, a greenish tinge. This circumstance induced me to discontinue the emetic, and prescribe an antispasmodic draught, to quiet the nervous agitation still existing. I gave, at the same time, four drachms of pure magnesia in a glass of tepid water, in order to neutralize the acid of the compound. I directed a ptisan of mallow-root

---

* *Observation sur un empoisonnement avec du bleu en liqueur; par M. Felix Genouville. Journal de Médecine, par Leroux, Mars* 1816. The composition is formed of nine parts of sulphuric acid, and one of indigo. "It is of a deep blue colour; thicker than sulphuric acid; of an oleaginous consistence; reddens tincture of litmus, and raises the temperature of water, on being mixed with it; evaporated to dryness, it disengages white heavy fumes of a penetrating odour, dependant on volatilization of a part of the sulphuric acid: the capsule contains a bright carbonaceous residuum. Heated with mercury, it is decomposed, and yields sulphuric acid gas, readily distinguished by its pungent smell, analogous to that of burnand liquorice to be taken during the day; and some spoonfuls of oil of sweet almonds every fifteen minutes. During the night, the patient felt severe pain about the pyloric orifice.

"On the morning of the 27th, I found her going on well. The pains were now felt only about the superior part of the œsophagus, and some whitish pellicles had been voided with the expectoration. She expressed a desire for food, and I recommended vermicelli. Three or four spoonsfull of it were swallowed with difficulty. In the evening, I prescribed an anodyne julep, which procured refreshing sleep during the whole night.

"28th. Violent pains in the stomach on awaking: a little jelly taken during the day with difficulty. Evening. Gastric pains very frequent. Anodyne potion repeated. Night very good.

"29th. Morning. Better: some pains yet in the stomach. Ptisan continued. Evening. Recurrence of pain augmenting in violence during the night."

30th. A little jelly taken for breakfast, only by drinking, after each spoonful, some of the ptisan. Evening. Still some gastric pain, more tolerable in the vertical position than the horizontal. Anodyne draught repeated with its wonted good effect."

31st. Pains much diminished. Some vermicelli had been taken in the morning with less difficulty. The erect posture productive of the same relief as yesterday. Ptisan, and almond oil continued. Evening. Some recurrence of pain; but the night tolerably good."

"February 1st. I found my young patient in a very comfortable state: she sat up the whole day. Towards evening, she again felt some pain; but the night passed more tranquilly than the last. From this period, Mademoiselle A. D. continued progressively to amend; and soon regained her pristine state of health.

---

ing sulphur. Lastly, if the acid be saturated by solution of caustic potash, it becomes green; and, evaporated in this state, dried, and calcined for fifteen minutes, leaves a carbonaceous substance, resulting from the decomposed indigo, and sulphate of potash. This latter may be dissolved in water, and converted into insoluble sulphate of barytes, by the addition of a sufficient quantity of any soluble barytic salt. This process is preferable to mixture of a barytic salt in the composition diluted with water." *Toxicologie par Orfila. Tome I. Partie II.*

PART

